# Local Anesthesia in Major Operations on the Head and Face

**Published:** 1914-03-15

**Authors:** Leo Eloesser

**Affiliations:** San Francisco


### LOCAL ANESTHESIA IN MAJOR OPERATIONS ON THE HEAD AND FACE.

---

BY LEO ELOESSER, M.D., SAN FRANCISCO.

---

The development of the methods of regional anesthesia and nerve blocking and their application to the head and face is the greatest advance that has been made in the surgery of these regions for some time. Their extensive practical application is a matter of the last three or four years. It is true that recent advances in general anesthesia, notably the administration of ether by catheterization of the upper air passages, the intratracheal insufflation of Meltzer and Auer and the intrapharyngeal insufflation of Crile by means of tubes passed through the nose have made operations in the cavities of the mouth and nose under a general anesthetic much easier and have done away with the distressing picture of a constant struggle between operator and anesthetist that was so common ten years ago. Still I think that you will agree with me that if we can avoid an inhalation method of anesthesia in these often long and difficult operations it is better. They predispose to postoperative pneumonias as it is—the act of swallowing is often impaired, it is sometimes impossible to avoid the aspiration of saliva or mucus and of the infectious contents of the mouth; if to these noxious agents, all favorable the to development of postoperative pneumonias and bronchitides, we add the insult of ether, the probability of serious damage to the lungs is much increased. It is not only the ether which is injurious; the very fact that the patient is unconscious, that he cannot cough up or spit out blood or saliva which may gather in his throat or gullet robs us of the aid of nature's most important reflexes to protect the lungs from harm. That these advantages of regional anesthesia over a general narcosis—the ability to cough up and spit out blood and saliva, absence of irritating vapors in the lungs—are not merely theoretical but are borne out by the facts we can see by the statistics of Braun, for example. In a paper published two years ago he cites 20 cases of major operations including the oral cavity, 8 resections of the maxilla, 12 cancers of the tongue and mouth with no operative mortality and with but two pulmonary complications. When we compare this with the previous mortality of some 20 per cent. we are inclined to regard regional anesthesia as little less than revolutionary in this province of surgery.

Permit me to refer you to this table taken from Hartel, (see Table at conclusion of article). By it you see that the cavities of the head may be divided roughly into three stories each innervated in the main by one of the trigeminal branches and each furnished with a conduit in which the innervating nerve runs and where it may be reached by an injection. The upper story corresponds to the first trigeminal branch and includes frontal, sphenoidal and ethmoidal sinuses and upper and anterior part of the nose. The nerve runs in orbid. The middle story corresponds to the second branch and includes the greater part of the nose, the antrum the naso-pharynx, and the mouth above the level of the teeth; the nerve runs in the pterygo-palatine fossa. The lower story corresponds to the third branch and includes the lower part of the mouth and some of the pharynx. The nerve runs in the infratemporal fossa. The innervation of the posterior part of this third story is supplemented by the sensory portion of the glosso-pharyngeal, vagus and upper cervical nerves, whose branches run in the bucco-pharyngeal tissues and in the soft parts of the floor of the mouth. The skin of the head on the other hand can not be divided into horizontal planes of innervation; its planes run more or less vertically, the anterior part corresponding to the first branch, the middle to the second, the rear to the third and to the upper cervical. When planning our nerve blocking therefore for a transverse incision we may cross all three branches in the skin and only one in the interior of the head, or vice versa.

A very simple way out of the study of the complicated innervation of these parts would be to block the whole trigeminal at once at the Gasserian ganglion itself. Then we should have an anesthesia of the whole face, which with the addition perhaps of a little simple infiltration of the upper cervical nerves would suffice for any operation in this region. This blocking of the ganglion has recently been carried out. I think that we may look upon it as the culminating point in the practice of regional anesthesia. After most careful anatomical study and measurements on 69 skulls Hartel described a method for reaching the ganglion. He anesthetises an elliptical area of the cheek, goes in opposite the second upper molar and marks on his canula the depth of the infratemporal plane (about 5-6 cm.) With one finger in the mouth as a guide he pushes his needle forward between the anterior border of the ascending ramus of the jaw and the maxillary tuberosity, going to the outside of the buccinator muscle. Viewed exactly from the front the canula should point to the pupil of the side on which the injection is made, viewed from the side towards the articular tuberosity of the zygoma. Feeling the way along the hard and smooth surface of the infratemporal plane the needle enters the oval foramen from the anterior outer side of the oval. A sudden decrease of resistance is felt by the operator and the patient has a sharp pain in the lower part of the face, the area innervated by the third branch. The needle is now pushed forward another centimeter and a half until the patient feels pain in the territory of the second branch. One cm. of 2 per cent. novocain-adrenalin solution is now injected and the resulting areas of anesthesia immediately tested. Successful anesthesia gives an anesthetic area of one side of the face. It cannot be denied that this method is not without a considerable risk, and that it is by no means ready for adoption in gerenal practice. The oval foramen is small and is situated at a depth of 5-6 cm. from the surface, the difficulty of striking so small an area at this distance is not slight; the proximity of such important structures as the brain and the carotid may make the slightest error of measurement a vital one. Hartel reports 39 successful cases, but among these he entered the dura and withdrew cerebro-spinal fluid on one occasion, with no ill results, it is true; on another he had a meningitis following his puncture. The possibility of such accidents in the hands of so experienced an operator would make us timid about attempting it unless most stringently indicated.

Should the future show a wide range of usefulness for this method of blocking the ganglion I think it quite probable that a safer way of access will be found where we can inject under the guidance of the eye, perhaps similar to the one first demonstrated by Harris some years ago where the ganglion was approached by an osteoplastic resection of the zygoma and retraction of the soft parts.

I have considered the most difficult and the least secure of these anesthetic methods first; I hope that this has not been bad policy; that it may not make you inclined to view the whole subject with doubt and to receive my further statements with a shake of the head. I wanted to bring this brilliant achievement of blocking the whole trifacial nerve with one c.c. of solution to your notice, even though I am not in a position to recommend it enthusiastically and in the lack of personal experience must advise caution.

The methods I propose to discuss I *can* recommend as I have tried them. They are not too difficult, and with ordinary care not dangerous. They may seem less simple than the injection of the whole ganglion because they force us to think out and to plan our procedures carefully. If we inject the whole ganglion there is no necessity for bearing in mind the precise innervation of the operative field;—the whole face is anesthetic and all we have to do is to go in—if we propose to block the separate trigeminal branches, however, we must plan exactly what we are going to do and have the innervation of our field at our finger's ends. It may not be necessary to block the whole branch at its exit from the skull, our operation may involve so limited an area that a mere peripheral injection may do. To know where to inject demands an exact knowledge of the innervation of the face and considerable forethought. It is impossible to formulate a rule of thumb for all cases, but reference to the table above will help with most of them.

The blocking of the first trigeminal branch, the ophthalmic, interests us less today. It will only come into question in oral operations including the upper part of the face. It is necessary for instance in resections of the maxilla involving the floor of the orbit. The lateral branches, the frontal, the lachrymal and the naso-ciliary are blocked without difficulty by an injection along the lateral orbital wall. The needle hugs the bone closely and feels it way to a depth of $4\frac{1}{2}$—-5 cm.; at this distance it impinges on the superior orbital fissure. Five c.c. of 2 per cent. novocain-adrenalin solution are injected at this point. As the injection is made completely outside the muscles of the eye, there is no danger of injury, provided we hug the bone closely as stated above. The injection for the ethmoidal nerves made along the inner wall of the orbit is often followed by a hematoma. This has never, so far as I know, produced disaster, still when possible I should prefer to block these nerves by the topical application of cocain to the nasal mucosa, aided, if necessary, by a subcutaneous injection across the bridge of the nose, as accomplishing the same result without the least risk. This only is passing. What concerns us mainly is the territory innervated by the two lower branches of the trigeminous. The whole territory of the second branch, the hard palate, the teeth, the upper lip, and the upper half of the mouth, the lower and posterior part of the nose, the naso-pharynx and the antrum may be rendered anesthetic by an injection into the second branch at its exit from the skull. The technique of the injection is not very difficult. The needle enters the skin directly behind the anterior angle of the zygoma and is pushed inwards and upwards. It traverses the masseter and impinges on the tuberosity of the maxilla. The operator now carefully feels his way along this hard smooth surface of bone. When the posterior end of the tuberosity is reached the needle usually slips easily into the pterygo-palatine fossa, sometimes, however, it impinges on the greater wing of the sphenoid. When this happens the needle is gently pointed downward a little. Sometimes it may even be necessary to withdraw it and enter the skin again at a point a little further back in the course of the zygoma. When the pterygo-palatine fossa is reached one feels a sudden loss of resistance, and pushing the needle forward gently strikes the nerve. The patient feels a sharp pain in the face and upper teeth, which is the signal for injection of 5 c.c. of 1-2 per cent. novocain-adrenalin solution. If a resection of the maxilla or other operation involving the internal maxillary artery is proposed a further 5 c.c. of solution is injected behind the mandible while withdrawing the needle. This is done in order that the adrenalin content of the solution may cause a contraction of the artery and lessen bleeding. By this method we get a complete anesthesia of the upper part of the mouth, cheek, and adjacent part of the nose. This method of injecting the second branch in the pterygo-palatine fossa is not difficult, yet it does require some practice, and I should certainly recommend its trial on the cadaver before attempting it on the patient. All the atlases and pictures and articles are not worth an hour spent in study and experiment on the cadaver. It is further advisable in the beginning to have a skull at hand when performing the injection. It gives us a feeling of security to be able to refer to this before carrying out an injection.

For many operations, all purely intraoral ones indeed, we do not need to block the whole second branch at all, the blocking of the upper dental and the palatine nerves will suffice. This is an easy procedure. The posterior superior alveolar nerves run across the tuberosity of the maxilla directly beneath the buccal mucosa before they enter the posterior alveolar foramen, and are accessible to the needle at this portion of their course. The anterior nerves do not run directly beneath the mucosa, but in the bone itself; the dividing layer of bone, however, is very thin and a submucous injection of a strong anesthetizing solution will diffuse through this layer and on into the nerves, provided we give it time enough. If, therefore, we begin at the frenulum of the upper lip and inject a line across the upper alveolar process working backwards to the tuberosity of the maxilla we can block all of the superior dental nerves and with them the pulps and the gums. The palatine part of the dental membrane is not affected by this injection, this part is served by the palatine nerves, which remain to be blocked, in order to render the whole alveolar contents anesthetic. This is a very easy matter; 2 ccs. of solution is enough. One c.c. across the posterior lateral part of the palate, will reach the anterior palatine nerves, and another c.c. at the mid-line anteriorly, and will block the naso-palatine nerves of Scarpa. So with a few ccs. of solution and with 15 or 20 minutes of time we can, by these three injections across the upper alveolar process, and across the front and rear of the hard palate, anesthetize the whole hard palate, the upper alveolar process and the upper teeth.

Now to the lower part of the mouth and the mandible. By blocking the third branch at the oval foramen we can make this whole territory anesthetic. We can do this in the same way as we block the second branch at the base of the skull, but it is a little more difficult, for we have no broad bony surface like that of the maxillary tuberosity to guide us. Our guide here is the bony plate of the external wing of the pterygoid, which lies at a depth of about 5 cm. from the skin. Close behind it lies the internal carotid, which we must avoid. We enter the skin at the middle of the lower border of the zygoma. The patient's mouth must be wide open, otherwise we shall collide with the coronoid process of the mandible. The needle is armed with a rubber cork, placed 5 cm. from its point. This marks the depth at which we expect to hit bone. It is slowly pushed forward in an exactly transverse direction until it strikes the pterygoid. The exact depth of this bone from the surface is now marked by sliding the rubber cork forward on the needle up to the skin. The needle is then withdrawn carefully until its point may be freely manipulated, usually, as far back as the subcutaneous tissue, and is pushed forward again, this time pointing about 1 cm. further backward. When the rubber marker reaches the skin the patient feels the characteristic pain in the third branch; if he does not the needle may be pushed forward a very little, not more than a half cm., on account of the nearby carotid. This will usually strike the branch. Several trials may, however, be necessary on account of the varying width of the pterygoid plate, which may force us to incline the needle backwards at a greater or less angle until we get behind it and can reach the foramen. Five ccs. of 2 per cent. novacain-adrenalin solution are used for the injection. Hartel of Berlin has recently described a method of approach from the cheek, similar to that described for the injection of the ganglion. He goes in at a point about opposite the second upper molar and passes his needle between maxilla and ascending ramus of the jaw in the maner previously described. This method offers a better bony guide than the transverse method, but is, I think, more dangerous as the internal maxillary artery lies in the path of the needle and the internal carotid just behind it.

Fortunately it is rarely necessary to have recourse to this somewhat exacting procedure of blocking the whole third branch. We have a peripheral point where we can reach both mandibular and lingual nerves with a single injection very easily, and secure practically the same effect; as though we had blocked the whole third branch. If we inject a strip across the inner side of the mandible high up, we reach both these nerves and get an anesthesia of all the lower teeth, the jaw, the lower lip, the upper part of the chin, the floor of the mouth and the tip of the tongue. This is one of the most elegant anesthetic procedures imaginable it is so simple and its effects so striking. About ½ cm. behind the last lower molar we feel the oblique line of the mandible, and just to the inside of this a little flat triangular field of bone, called by Braun the retromolar triangle. This is our guide for the injection; we enter the mucosa here, keeping the needle parallel to the plane of the lower teeth and its point well against the inner bony surface of the jaw. We deposit ½ cc. of solution directly beneath the mucosa; this blocks the lingual nerve, then feeling our way backwards along the bone we keep on injecting, and proceed in this way for 2-3 cm. We have now injected a strip of solution across the path of the mandibular nerve. In about 5 minutes the lingual nerve is anesthetized, and in another 10, the mandibular. For resections of the jaw or for attacks on the submaxillary fossa we need an anesthesia of the upper cervical nerves. These may be blocked by an injection along the posterior border of the sterno-cleido-mastoid muscle, or they may be anesthetized *in loco* by a subcutaneous injection along the jaw itself.

I shall not tire you by enumerating further methods, such as the anesthesias for the skin of the face by injections at the supra and infraorbital foramina, and all the varied sites for anesthesias in the cervical region. Once familiar with the general principles and methods of regional anesthesia one works out these methods for oneself.

It may not be out of place here to discuss the general principles on which this regional anesthesia is based. The key to the whole procedure is *nerve-blocking* as against strictly *local anesthesia.* If we run our needle along in the skin and deposit our anesthetic at the line where we are going to use the knife we have a purely local anesthesia. If we go around this area, injecting an imaginary rhombic figure we block the nerves leading to the field of operation, and are no longer producing a local anesthesia in the strict sense of the word, but a regional one. This is of great value in that it enables us to block off considerable areas with very small amounts of solution. Suppose the nerve supply all comes from one point, then by injecting this point we can block off a large field with one drop of anesthetic, provided we get that drop in just at the right spot. Or suppose the nerves come from several points, then we must inject the several sources of supply. If we were to infiltrate the whole field, injecting until the territory was everywhere permeated with novocain, we might need many ounces of solution.

This nerve-blocking or regional anesthesia has been made possible by the brilliant discovery of Braun, whose importance we can scarcely overestimate, of combining the anesthetic drug with the active principle of the adrenal gland, adrenalin or some similar preparation. Before this discovery it was impossible to block nerves simply because the drug would be absorbed and disappear from the site of injection before it had *time* to penetrate the nerve trunks. The addition of adrenalin contracts the blood vessels at the site of injection, the anesthetic drug is no longer absorbed by the blood stream, it remains where its effect is wanted and it has time to diffuse through the thickness of the nerve trunks before disappearing from the place where our needle has deposited it. It takes time for a drug to diffuse through the thickness of a nerve —5 or 10 or 15 minutes. The nerves are not microscopic filaments, they have a very considerable thickness and body, many of the nerves of the head are larger than a good-sized round match. It takes time for a drug to penetrate their resistant sheath, and if it is absorbed before it *does* penetrate, then of course we get no anesthetic effect. So that this idea of Braun's of adding adrenalin contracting the blood vessels and making his anesthetic stay where he puts it and wants it was really the starting point for all regional anesthesia.

As to the drugs and the general technique. We can get along very well with but two local anesthetics—first, novocain. This is by far the most important. It is very little toxic, very much less so than cocain, so that we are free to use large quantities, 2 grams and more, corresponding to about 6 ounces of 1 per cent. solution have been injected without ill effects. It is non-irritating, produces no necrosis of the tissues, and although it suffers somewhat by boiling it may be sterilized by boiling up once and cooling rapidly. I think that almost everyone is agreed that it is an almost ideal drug for the purpose. Even the French, who long held to stovain, have conceded its superiority, and it has almost completely supplanted eucain, alypin, stovain, and all other similar compounds. It is most conveniently used in the tablet form. These tablets are put up in various strengths by the factory at Hochst in Germany. The factory says that they are sterile. One of them dropped into sterile salt solution dissolves quickly and gives us a fresh solution whose concentration we can vary at will. The tablets already contain suprarenin so that a further addition of adrenalin is unnecessary. Now although the factory says that the tablets are sterile, bacteriological tests have proven that they are not, and it may be more prudent to boil up the solution quickly and to cool it rapidly under a tap of water. I should certainly advise doing this if the solution is to be injected into a place where even a slight infection may do grave harm, such as the injection into the Gasserian ganglion. There is no doubt, however, that the anesthetic power of the drug suffers considerably by boiling. I have usually not boiled the solution, and have seen no ill effects.

The second indispensable drug is cocain. We cannot do without it for topical applications to the mucous membranes. Its anesthetic effect is stronger than that of novocain, but it is quite toxic, and has an unaccountable action on persons peculiarly susceptible. A number of deaths have occurred from its use, even in small amounts, and it is for this reason that novocain has entirely replaced it for subcutaneous injection. It is besides irritating to the tissues, and cannot stand boiling.

Both of these drugs are used in connection with the active principle of the adrenal gland, which for brevity's sake I have termed adrenalin throughout this paper, not meaning thereby any one particular pharmaceutical product. The synthetic adrenalin, suprarenin, is said to be capable of sterilization by boiling if a trace of hydrochloric acid be added to the solution. The adrenalin which is extracted from animal glands cannot be boiled. The use of a small Record or a Luer syringe with a fine needle is advisable. Always use a small syringe, not over one or two cc. capacity. By using a small syringe we get a more even distribution of our solution and are much less likely to use excessively large quantities than if we use a large one. Always follow the old rule to keep both needle and piston of the syringe moving at the same time, that is, do not inject unless moving the needle forward or retracting it in the tissues. In this way we get the solutions evenly distributed instead of depositing large amounts in one place and nothing in another, and run no risk of injecting the anesthetic into a vein or artery, should the needle happen to puncture one.

A word as to the patient. We should explain the procedure fully to the patient, and gaining his confidence assure him that while there will be *feeling* in the anesthetized parts there will be no pain. That merely the first prick of the needle will hurt and nothing more. We should ask him to let us know if he does feel pain and assure him if he does we will stop. And we should stop. STOP, and stop immediately. Once the patient feels that something is being done that is causing him pain he becomes frightened and nervous and we can do nothing further with him. Stop, make further injections, get a good anesthesia and a quiet patient, or if you can't, go over to a general anesthetic. There is no more distressing sight than that of an operator struggling with himself, with his operation and with a frightened, wretched patient under an insufficient local anesthesia. It is impossible to do good work under these conditions and the infliction on the patient is inexcusable.

Children, or adults not amenable to reason, nervous or frightened women and men should have a general anesthetic. There is no use in trying to talk a reluctant patient into having a local anesthetic, if he is afraid of it he should have a general one. In major operations. it is good to supplement the local anesthetic by a mild general one, in some cases an injection of morphine ¼ gr. and scopolamin 1-100 gr. given one-half to three-quarters of an hour before operation is almost indispensable; 5 grs. of veronal a few hours before is also useful.

1. Blocking of the whole Gasserian ganglion has been successfully practiced.

2. Blocking of the second and third tringeminal branches at their points of exit from the skull is easier and of more extensive application.

3. Peripheral injections for anesthesia of more limited areas are easily carried out and are useful. Of particular dental interest are: the anesthesia of the second branch of the maxilla and the palate, and of the third branch at the inner surface of the jaw.

4. Novocain is the anesthetic of choice. It is always to be used in combination with some preparation of the adrenal gland.

5. Reluctant or frightened patients, children and adults not amenable to reason are to be excluded from local anesthesia.

---

TABLE. (After Hartel).

*Sensory Innervation of Skin of Face by Trigeminus.*

| BRANCH | NERVE | INNERVATED AREA |
|---|---|---|
| I. | Lachrymal | Outer canthus. |
| | Frontal | Upper eyelid, forehead, inner canthus. |
| | Naso-ciliary | Tip of nose, inner canthus. |
| II. | Zygomatic | Temple, zygomatic region. |
| | Infraorbital | Ala of nose, lower eyelid, anterior. part of cheek, upper lip. |
| III. | Buccinator | Angle of mouth. |
| | Auriculo-temporal | Anterior part of ear, temple, cheek. |
| | Inferior alveolar (mental) | Lower lip, chin. |

*Sensory Innervation of Mucous Membranes.*

1. *Nose and Accessory Sinuses.*

| MAIN BRANCH | NERVE | SENSORY BRANCH | INNERVATED AREA. |
|---|---|---|---|
| *a) Nose.* | | | |
| I. | Naso-ciliary | Ant. ethmoidal (ant. nasal) | Upper anterior nose. |
| II. | Spheno-palatine (nerve & ganglion) | Sup. & inf. post nasal | Remainder of nose. |

*b)* *Sinuses.*

| | | | |
|---|---|---|---|
| I. | Naso-ciliary | Post. ethmoidal | Sphenoidal sinus<br>Post. ethmoidal sinuses |
| | " | Ant. ethmoidal | Ant. ethmoidal sinuses<br>Frontal sinus |
| II. | Spheno-palatine | Nasal branches | Highmore's antrum |
| | Infraorbital | Post., med., & ant. superior alveolar. | |

2. *Oral Cavity.*

| MAIN BRANCH | NERVE | SENSORY BRANCH | INNERVATED AREA |
|---|---|---|---|
| II. | Infraorbital | Super. alveolar | Upper teeth, buccal side of gums |
| | | Sup. labial | Mucosa of upper lip |
| | Spheno-palatine ganglion | Naso-palatine<br>Palatine | Palatal side of alveolar peridentium and gums, hard and soft palate. |
| III. | Inf. alveolar | Dental | Lower teeth and gums. |
| | | Mental | Mucosa of lower lip. |
| | Lingual | Sublingual | Lingual side of gums of lower incisors. |
| | | Lingual | Tongue up to foramen cecum. |
| | | Faucial isthmial | Part of tonsil. |
| | Buccinator | Buccinator | Buccal mucosa. |

3. *Pharynx and Larynx.*

| MAIN BRANCH | NERVE | SENSORY BRANCH | INNERVATED AREA |
|---|---|---|---|
| II. | Sepheno-palatine ganglion | Pharyngeal | Region of Eustachian tube. |
| III. | Lingual | Faucial isthmial. | Part of tonsil. |
| | Glosso-pharyngeal | Pharyngeal | Pharynx (together with vagus) |
| | | Tonsillar | Tonsil, palatine arches. |
| | | Lingual | Tongue behind foramen cecum. |
| | Vagus | Pharyngeal | Pharynx. |
| | | | Base of tongue near epiglottis. |
| | Superior laryngeal | Internal branch | Entrance of larynx. |
| | | External branch | Lower part of larynx. |

*—Pacific Dental Gazette, February, 1914.*

